# Unfolding the role of placental-derived Extracellular Vesicles in Pregnancy: From homeostasis to pathophysiology

**DOI:** 10.3389/fcell.2022.1060850

**Published:** 2022-11-21

**Authors:** Miguel A. Ortega, Oscar Fraile-Martínez, Cielo García-Montero, Alberto Paradela, María Asunción Sánchez-Gil, Sonia Rodriguez-Martin, Juan A. De León-Luis, Claude Pereda-Cerquella, Julia Bujan, Luis G. Guijarro, Melchor Alvarez-Mon, Natalio García-Honduvilla

**Affiliations:** ^1^ Department of Medicine and Medical Specialities, Faculty of Medicine and Health Sciences, University of Alcalá, Alcala de Henares, Spain; ^2^ Ramón y Cajal Institute of Sanitary Research (IRYCIS), Madrid, Spain; ^3^ Cancer Registry and Pathology Department, Principe de Asturias University Hospital, Alcala de Henares, Spain; ^4^ Proteomics Core Facility, CNB-CSIC, Madrid, Spain; ^5^ University Defense Center of Madrid (CUD), Madrid, Spain; ^6^ Service of Pediatric, Hospital Universitario Principe de Asturias, Alcalá de Henares, Spain; ^7^ Department of Obstetrics and Gynecology, University Hospital Gregorio Marañón, Madrid, Spain; ^8^ Health Research Institute Gregorio Marañón, Madrid, Spain; ^9^ Department of Public and Maternal and Child Health, School of Medicine, Complutense University of Ma-drid, Madrid, Spain; ^10^ Unit of Biochemistry and Molecular Biology, Centro de Investigación Biomédica en Red en El Área Temática de Enfermedades Hepáticas (CIBEREHD), Department of System Biology, University of Alcalá, Alcala de Henares, Spain; ^11^ Immune System Diseases-Rheumatology, Oncology Service an Internal Medicine, Centro de Investigación Biomédica en Red en El Área Temática de Enfermedades Hepáticas (CIBEREHD), University Hospital Príncipe de Asturias, Alcala de Henares, Spain

**Keywords:** extracellular vesicles, placental-derived extracellular vesicles, normal pregnancy, pre-eclampsia, gestational diabetes mellitus, obstetric complications, placentation, decidualization

## Abstract

The human placenta is a critical structure with multiple roles in pregnancy, including fetal nutrition and support, immunological, mechanical and chemical barrier as well as an endocrine activity. Besides, a growing body of evidence highlight the relevance of this organ on the maternofetal wellbeing not only during gestation, but also from birth onwards. Extracellular vesicles (EVs) are complex macromolecular structures of different size and content, acting as carriers of a diverse set of molecules and information from donor to recipient cells. Since its early development, the production and function of placental-derived EVs are essential to ensure an adequate progress of pregnancy. In turn, the fetus receives and produce their own EVs, highlighting the importance of these components in the maternofetal communication. Moreover, several studies have shown the clinical relevance of EVs in different obstetric pathologies such as preeclampsia, infectious diseases or gestational diabetes, among others, suggesting that they could be used as pathophysiological biomarkers of these diseases. Overall, the aim of this article is to present an updated review of the published basic and translational knowledge focusing on the role of placental-derived EVs in normal and pathological pregnancies. We suggest as well future lines of research to take in this novel and promising field.

## 1 Introduction

### 1.1 A global view on human placenta

The human placenta is formed after the implantation process of the zygote (in form of blastocyst) to the endometrium. Once the blastocyst is attached, two layers are formed: an inner layer, responsible for the formation of the embryo and an outer layer designed as trophectoderm, which ultimately will lead to the process of placentation (Wang et al., 2017). Simultaneously, the stromal cells present in the maternal endometrium will start the process of decidualization, remodeling the uterus and promoting implantation and placentation success (CY et al., 2010).

The cells present in the trophectoderm are named trophoblasts and they will form the fetal portion of the placenta, whereas the decidual layer will be the maternal one. Trophoblasts will migrate from the trophectoderm to the endometrium, invading the epithelium and maternal blood (Staud and Karahoda, 2018). This process is started by the syncytiotrophoblasts (STBs), which are oligonucleated cells formed by the fusion of mononucleated trophoblasts. The other main cellular component of the placenta are the cytotrophoblasts (CTBs), composed by the remaining mononucleated trophoblasts (Huppertz, 2008). STBs will contact the maternal blood at early stages whereas CTBs will migrate from the fetal to the maternal portion of the placenta, turning into endovascular extravillous trophoblasts (eEVTs) and interstitial trophoblasts (iEVTs). iEVTs will be located in the endometrial decidua, whereas eEVTs will be critical for the remodeling of spiral arteries in the maternal endometrium (Huppertz, 2018). The process of placentation is completed with the development of placental villi. In the first trimester of pregnancy, a system of villous trees is formed as well as the intervillous space, in which the maternal blood is shed (Aplin et al., 2018). An outer layer of STBs together with inner CTBs will be responsible for the formation of primary villi. Next, extra-embryonic mesodermal cells will invade the primary villi, forming a mesenchymal core and transforming them into secondary villi. These represent a major source of extravillous trophoblasts (EVTs), either iEVTs and eEVTs (Castellucci et al., 2000; Huppertz, 2008). Finally, fetal cappilaries appear in the core of the secondary villi, concluding the process with the formation of tertiary villi (Turco and Moffett, 2019).

The development and growth of the human placenta is remarkably rapid. In non-pathological conditions, their weight increases from 50 g at 10–12 weeks to the 500–600 g that the placenta typically reaches at delivery (Fadl et al., 2017). In this period, tertiary villous trees proliferate, and different subtypes will form: 1) Mesenchymal villous trees, precursor of the other villous trees, essential for the endocrine activity of the placenta; 2) Immature intermediate villi, representing a branching of mesenchymal villous trees; 3) Stem villi, with structural support function; 4) Mature intermediate villi, highly vascularized, fulfilling maternofetal exchange and leading to the formation of the 5) terminal villi, which are the most mature form of the placental (or chorionic) villi, mediating the transfer of oxygen/carbon dioxide, electrolytes, and nutrients between the mother and fetus (Benirschke et al., 2006; Huppertz, 2008; Wang and Zhao, 2010). One or more fetal villous trees are grouped in structures known as cotyledons, which contain a fetal artery and a vein, and are separated by connective tissue and irrigated by one or more maternal spiral arteries (Barker et al., 2013). Although differences exist, there are some critical cellular groups that are located in these structures in addition to the aforementioned STBs and CTBs. These include different cell populations derived from mesenchymal cells (including fibroblasts, myofibroblasts, or smooth muscle cells), Hofbauer cells, which are local macrophages populations derived from mesenchymal cells or recruited from maternal blood, fetal vessels and different extracellular materials collectively known as fibrinoids, regulating cellular behavior in the placenta (Huppertz, 2008; Aplin et al., 2018). In addition, there is a plethora of maternal immune populations present in the endometrial decidua, including decidual natural killer cells (dNKs), dendritic cells (DCs), T cells and macrophages (Tong and Abrahams, 2020). Trophoblasts are semi-allogenic cells, combining both maternal and paternal genetic backgrounds (JK, 2008). Interestingly, they exert critical immunomodulatory functions, modulating the maternal immune response and avoiding the rejection of the embryo and placenta. In addition, the inflammatory environment provided by the combined action of the maternal immune system, decidual cells and trophoblasts is critical for the development of the placenta during the first trimester (Aaltonen et al., 2005; Burton and Jauniaux, 2015; Goldstein et al., 2020)**.** Conversely, the second trimester is mostly an anti-inflammatory stage whereas the third trimester and specially the delivery process are pro-inflammatory (Mor et al., 2011).

In summary, the placenta is a complex structure with a pivotal role during pregnancy, orchestrating multiple physiological processes critical for pregnancy success. This organ represents a structural, immunological and chemical barrier between the fetus and the mother, simultaneously allowing the exchange of oxygen, nutrients and various metabolites between the mother and the fetus. In addition, the placenta performs essential endocrine functions (Gude et al., 2004; Lewis et al., 2012; Larqué et al., 2013). The importance of the placenta is illustrated by its central role in the genesis and development of various obstetric pathologies. (Audette and KingdomScreening, 2018; Travaglino et al., 2019; Ortega et al., 2021). Besides, this organ is a remarkable epigenetic modulator both for the fetus and the mother, influencing the maternofetal wellbeing after the delivery (Burton and Fowden, 2015; Burton et al., 2016).

### 1.2 Extracellular vesicles

Extracellular vesicles (EVs) are carriers of different biological molecules, being relevant actors in both physiological and pathological processes (Yáñez-Mó et al., 2015; Kalra et al., 2016). For these reasons, there is a growing interest in the research and potential applications of EVs (Couch et al., 2021). EVs are produced by all cell types (Akers et al., 2013) and act in different recipient cells in an autocrine, paracrine or endocrine manner, modulating several cellular processes (Zaborowski et al., 2015).

Traditionally, EVs have been divided in three main groups: exosomes, ectosomes or microvesicles, and apoptotic bodies. They differ in their size, biogenesis, content, release and biological functions (Yáñez-Mó et al., 2015). According to size, exosomes are the smallest EVs (∼30–100 nm), followed by microvesicles (100–1,000 nm) and apoptotic bodies (ranging from 1 to 5 µm), with some degree of overlapping between the different categories (Willms et al., 2018). Regarding the biogenesis, exosomes are produced by the late endosomes, also known as multivesicular bodies (MVBs). During the formation of MVBs, there are a set of proteins and cellular complexes such as ESCRTs (endosomal classification complexes required for transport) or tetraspanins which incorporates in a highly selective way different products which are recycled from the plasma membrane or directly incorporated from the cytosol, being enclosed in intraluminal vesicles (ILVs) (Zhang et al., 2019). Then, the late endosomes can either fuse with lysosomes and be degraded or they can move to the plasma membrane. After fusion of the MVB with the cell membrane, the ILVs are released to the extracellular space, being designed as exosomes (Ortega et al., 2022a). Microvesicles are formed by direct budding of the plasma membrane, as a result of coordinated work between membrane phospholipids and the contractile action of the cellular cytoskeleton (Kalra et al., 2016). This process is coordinated by different complexes such as ATP-binding cassette transporter 1 (ABCA1), and other molecules also involved in the biogenesis of exosomes like the aforementioned ESCRT, ARF6 and phospholipase D2 (PLD2), although their mechanisms of action are different (Tricarico et al., 2017). In the case of apoptotic bodies, their biogenesis is linked to programmed cell death or apoptosis in a three stage process: 1) plasma membrane blebbing; 2) formation of thin apoptotic membrane protrusions and 3) fragmentation into individual apoptotic bodies (Akers et al., 2013). For a detailed view of the biogenesis of the different EVs, see (Kang et al., 2021).

EVs content includes a broad spectrum of lipids, proteins and nucleic acids, including different types of RNA and DNA (Zaborowski et al., 2015). Apoptotic bodies can also contain micronuclei, portions of cytoplasm, degraded proteins, chromatin remnants, DNA fragments, large macromolecular complexes and even intact cell organelles (Battistelli and Falcieri, 2020). Exosomes and microvesicles interact and are recognized by specific cell membrane receptors. This process triggers the internalization of the exosomes and microvesicles inside the cell and the release of their content within the cell. They can also be internalized by phagocytosis, macropinocytosis, or different types of micropinocytosis, giving rise to early endosomes. Apoptotic bodies are phagocytosed by different cells, especially macrophages in the process of spherocytosis (Kourtzelis et al., 2020). Once the EVs enter in the recipient cell, they can be recycled back to the plasma membrane and released, or they can be directed to MVBs, where they will fuse with lysosomes for degradation or their content will be released in the cytosol, nucleus, or ER, where they will exert their cellular functions (Ortega et al., 2022a).

EVs are produced by many cell types, and especially in active tissues and organs, as it is the case of human placenta (Tersigni et al., 2022). In recent years, a growing number of studies have studied the different functions of EVs during pregnancy, at different gestational stages and emphasizing their role in pathophysiological processes (Das and Kale, 2020; Nakahara et al., 2020). The aim of this work is to review the role of placental-derived EVs (PEVs) in normal and pathological pregnancies, as well as future directions to take in this novel and promising field of research.

## 2 Placental-derived extracellular vesicles. Relevance and types

PEVs are essential to understand how pregnancy is regulated. Their effects are both local and systemic, orchestrating multiple processes. Indeed, there is evidence that PEVs are pivotal mediators of the maternofetal interplay, affecting different fetal and maternal tissues and organs (Nakahara et al., 2020). STBs appears to be a major source of PEVs, although CTBs, mesenchymal cells, EVTs and other cell groups from the placenta are also involved in their production (Kupper and Huppertz, 2022)**.** The amount, biogenesis, content and biological functions of the PEVs differ significantly according to the trimester of gestation and the physiological status of the feto-placental unit (Zhang et al., 2020; Condrat et al., 2021). Many studies have shown that the placenta-specific enzyme placental-type alkaline phosphatase (PLAP) is a relevant molecular marker of placental and fetal EVs, being widely used for the characterization and classification of PEVs (Mincheva-Nilsson and Baranov, 2014). Placental EVs include exosomes, microvesicles and apoptotic bodies (Nakahara et al., 2020). PEVs can be detected in maternal blood after 6 weeks, when the blood flow into the intervillous space (∼10 weeks gestation), is not yet established. However, the mechanism supporting the transfer of PEVs into maternal blood at this early stage is not known (Tannetta et al., 2017). In the first trimester of pregnancy, the number and concentration of PEVs appears to increase steadily (Sarker et al., 2014). This trend is maintained during the whole pregnancy, and several works have described that the concentrations of PEVs are approximately 20 times greater after 28 weeks of pregnancy compared to non-pregnant women (Sabapatha et al., 2006). The highest exosomal concentration in maternal blood is reported at term (Salomon et al., 2013). It is equally important to remark that there are some notable challenges to face when working with PEVs. Firstly, for sample collection it is frequent to use minimally invasive fluid samples in humans, whereas *in vitro* or animal models can also be used to study PEVs (Block et al., 2021). All of them may present critical advantages and disadvantages. For instance, there are different types of *in vitro* models like primary trophoblast cultures or placental cultures which can allow to define a cell type-specific secretory profile in response to an experimental manipulation; permitting to confirm a placental origin of the EVs (Fitzgerald et al., 2018). However, there are some important limitations regarding the viability or the gene and protein expression profiles, which can be compensated with the use of animal models, allowing the control of experimental factors while facilitating the access to large cohorts, rigorous sampling, the use of the animal as their own control or the possibility of driving transgenerational studies (Block et al., 2021). Nevertheless, the results obtained in animal studies might not be totally extrapolated to humans, and there are less studies in comparison to samples obtained from maternal fluids, which present larger database and is readily accessible. There are different isolation techniques currently used, such as size exclusion chromatography plus immunosorbent procedure against PLAP, microfluidic chip and ultrasonic waves as well as different kits; however, sequential centrifugations at low speeds followed by ultracentrifugation at 100.000 x g (or above) are the standard protocol for isolating PEVs, with different modifications based on this approach (Burkova et al., 2021). These techniques are not exempt from failures, and sometimes it is difficult to distinguish between different types of PEVs, due to their overlapping size. Besides, there are also some studies hesitating about the general use of ultracentrifugation, as this may result in aggregation of EVs (Yuana et al., 2015). Thus, there is a need for an standardized protocol for the study of PEVs. Storage of EVs generally extracted from plasma or other human fluids can be achieved up to 1 year following a freezing process, with insignificant changes in the composition (Yuana et al., 2015). The analysis of the content of PEVs represents another challenge to face, as if the sample studied is insufficiently purified or the isolation process has not been successfully, preparations containing other non-exosomal vesicles and co-isolated proteins may be analyzed (Burkova et al., 2021).

**FIGURE 1:**
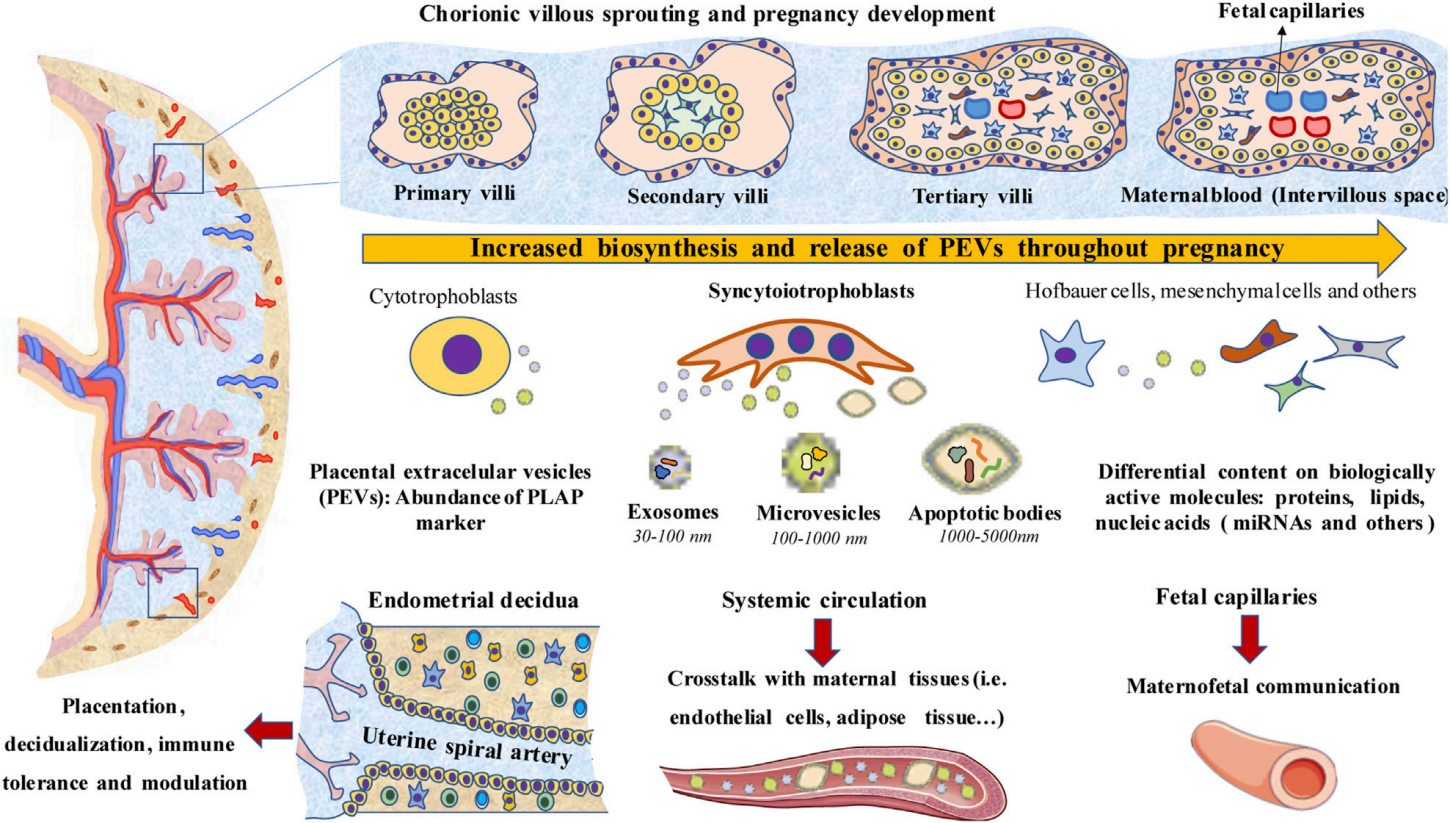
A general overview of PEVs. Biosynthesis and release of PEVs increase as pregnancy progresses along with the development of the placental structures. Syncytiotrophoblasts, which are in contact with maternal blood (Intervillous space), are a major source of PEVs, although cytotrophoblast, Hofbauer cells, mesenchymal cells, extravillous trophoblasts and others also produce them. PEVs are essential for the interplay between the placenta and endometrial decidua, modulating both the placentation and decidualization processes. PEVs also modulate the immune system, the inflammatory response and immune tolerance. PEVs reach systemic circulation or enter through fetal capillaries into the fetal circulation, playing a key role in the crosstalk between the placenta with maternal and fetal tissues.

Figure 1 summarizes a global overview of PEVs main characteristics, including main cell sources, levels and potential implications. We will summarize in this section the main features and implications of the different PEVs in non-pathological pregnancies.

### 2.1 Placental exosomes

Placenta-derived exosomes (pEXO) are released by different cell types in the human placenta, and carry significant amounts of growth factors, DNA fragments, miRNAs, and messenger RNAs (Jin and Menon, 2018). In addition, pEXOs present a differential profile of phospholipids and proteins in comparison to placenta-derived microvesicles and apoptotic bodies. Indeed, pEXOs appears to have greater stability and reduced fusogenic properties (Ouyang et al., 2016).

The content, biogenesis and release of pEXOs is closely regulated by the placental microenvironment. For instance, several studies have reported an increased exosome release by trophoblast cells under hypoxic conditions or in the presence of high glucose levels (Mitchell et al., 2015). Different mechanisms describe the effect of pEXOs interaction with both the fetus and the mother, critically influencing several developmental events. Most of the studies regarding the effect of pEXOs in physiological pregnancies focus on their immunomodulatory aspects. As aforementioned, the induction of an immune tolerance by the maternal immune cells is critical to prevent the rejection and ensure an adequate development of the semi-allogeneic fetus. In this line, previous studies have found that pEXOs present central immunomodulatory proteins such as human leukocytic antigen (HLA)-G5, B7-H1 and B7-H3 at early and term placental explants (Kshirsagar et al., 2012). The former was only secreted in early explants-derived pellets but not at term stages, although CTBs could sustain the production of HLA-G5 during pregnancy. The expression of different types of HLA during pregnancy is critical for ensuring immune tolerance in pregnancy, and several obstetric complications are characterized by an abnormal expression of HLA molecules (Tersigni et al., 2020). Hence, pEXOs are critical for promoting this immune tolerance, especially at early and late stages, preventing potential complications. Other results report that trophoblasts-derived exosomes could enhance macrophage migration in a dose dependent manner, leading to an increased production of interleukin (IL)-1β, IL-6, Serpin-E1, granulocyte colony-stimulating factor, granulocyte/monocyte colony-stimulating factor, and tumor necrosis factor-α (TNF-α) (Atay et al., 2011). In pregnant mice, pEXOs appears to interact specifically with maternal lungs and liver, where interstitial macrophages uptake these EVs. However, the physiological consequences of this fact is not fully understood (Gerosa et al., 2016). Macrophages are actively involved in trophoblast invasion, tissue and vascular remodeling during early pregnancy, presenting a different polarization according to the environment: M1 (Pro-inflammatory) and M2 (Anti-inflammatory) (Jena et al., 2019). The ratio between M1 and M2 populations is essential for pregnancy success, although it can vary across pregnancy, with an increased M1 polarization during the peri-implantation state, mixed M1/M2 profiles from the attachment to the development of the placenta and a shift towards M2 polarization until term labor, where an increase of M1 is again reported (Yao et al., 2019). In this sense, a recent study conducted by Bai et al., (2022) described that pEXOs can regulate circulating monocytes, driving to a M2 polarization. This effect was attributed to a microRNA present in these exosomes, miR-29a-3p, which promoted the expression of programmed cell death ligand-1 (PDL-1), a well-known surface receptor that suppresses the adaptive immune system. Thereby, pEXOs can modulate macrophage behavior and its polarization, although further studies are warranted to deepen on potential mechanisms involved in these events. pEXOs modulate NK cells as well, as shown in previous works. In fact, it has been described that they can be internalized both *in vitro* and *in vivo*, modulating the expression of several markers, inducing CD56^dim^ NK cells and apoptosis, and promoting the development of decidual NK cell-like phenotype (Bai et al., 2021). Interestingly, NK, macrophages and activated T CD8 cells express high levels of NK group 2 member D (NKG2D) and it has been demonstrated how pEXOs carries NKGD2 ligands (i.e. UL-16 binding proteins (ULBP)) and MHC class I chain-related (MIC) proteins A and B that exert an immunosuppressive function in these cells (Hedlund et al., 2009)**.** Besides, pEXOs equally content miR-517a-3p, targeting PRKG1 in NK, hence influencing in the function of these cells (Kambe et al., 2014). Other mechanisms by which pEXOs modulates several immune populations include the presence in these PEVs of PDL-1 or PDL-2 and proapoptotic ligands such as TRAIL and FasL (Mincheva-Nilsson, 2021)**.** Collectively, the modulatory role of pEXOs in NK cells and other populations denote the relevance of these EVs during physiological pregnancy.

In addition to its immunomodulatory role, pEXOs fulfill different functions in physiological pregnancies. For instance, previous works denoted that pEXOs appears to induce the mechanisms of defense against different viruses and pathogens (Delorme-Axford et al., 2013; Bayer et al., 2015). Different miRNA members of the chromosome 19 miRNA cluster (C19MC) seem to be potentially involved in these processes, protecting the mother and fetus against infectious diseases. Likewise, these components are relevant for the placentation and vasculogenesis processes. Thus, exosomes isolated from placental mesenchymal stem cells induce endothelial cell migration and vascular tube formation (Komaki et al., 2017). Likewise, in non-pathological pregnancies, the effect of pEXOs on endothelial cell migration is higher in the first trimester with respect to the second and third trimester (Salomon et al., 2014a). Under hypoxic conditions cytotrophoblast-derived exosomes appear to induce EVTs migration (Salomon et al., 2013). Non-exacerbated hypoxia is crucial in normal pregnancies, especially during the process of placentation (Soares et al., 2017). In turn, it seems that pEXOs secreted by EVTs are critically involved in the uterine spiral artery remodeling at early stages (Salomon et al., 2014b). Beyond this, previous works have defined the relevance of pEXOs for fetal development and health. Miranda et al. (Miranda et al., 2018) reported that quantification of pEXOs in maternal plasma at third trimester are great indicators of fetal growth, representing a potential reflection of placental function. More detailly, they observe that there was a significant positive correlation between the ratio of placental derived to total exosomes (PLAP^+ve^ ratio) and birth weight percentile. In this line, some authors have argued that PLAP appears to be essential to assist the transfer of maternal IgG to the fetus at the placenta surface, aiding to stimulate DNA synthesis and cell proliferation in fetal fibroblasts, improving its survival (Jin and Menon, 2018). To this observation, it must be added the different components previously mentioned, aiding to explain the relevance of pEXOs in the development and physiological status of the fetus and the mother.

### 2.2 Placental microvesicles and apoptotic bodies

Placental microvesicles have been less studied in pregnancy. STBs are a major source of these microvesicles, especially due to the shedding of their microvilli or in association with syncytial nuclear aggregates (SNAs), although the mechanisms or regulation of this process are not fully understood (Chamley et al., 2014; Tong and Chamley, 2015). PLAP can be used as well to tag placenta-derived microvesicles (Dragovic et al., 2015). CTBs exposed to the maternal blood following denudation of the syncytiotrophoblast or extravillous trophoblasts can also be involved in microvesicles release (Tong and Chamley, 2015). Similar to pEXOs, placental microvesicles are also critical modulators of the immune system. It has been demonstrated that circulating microvesicles in the second trimester of gestation present high levels of immunosuppressive TGF-β1 and IL-10, with an enhanced caspase-3 activity in CD56^dim^ NK cells (Nardi et al., 2016). Likewise, syncytiotrophoblast-derived microvesicles (STBMs) isolated in the third trimester of pregnancy bind to B cells and monocytes, modulating the expression of several cytokines involved in type 2 immunity and increasing the expression of IL-6 and TNF-α with respect to non-pregnant women (Southcombe et al., 2011). Likewise, IL-1β is more abundant in placental microvesicles during the first trimester of pregnancy, as well as in certain placental diseases. This interleukine is relevant for the immune cell activation and responsiveness to bacterial lipopolysaccharide (LPS) (Holder et al., 2012). Interestingly, the antiviral activity of placental microvesicles is less relevant with respect to exosomes although they have a modulatory role in the angiogenesis process (Tannetta et al., 2013).

Apoptosis is essential for normal placental development, as trophoblast apoptosis increases with placental growth and pregnancy progresses (Athapathu et al., 2003). It has been hypothesized that this process involves the clustering of apoptotic nuclei and liberation of this material into the maternal circulation (Sharp et al., 2010). As previously mentioned, placental microvesicles and apoptotic bodies differ from pEXOs in phospholipid and a protein profiles, although the miRNAs cargo is similar (Ouyang et al., 2016). Tong et al., (2016) demonstrated in an *ex vivo* culture of first trimester placenta that microvesicles carried more proteins compared to exosomes and apoptotic bodies. Likewise, the majority of proteins are shared by all EVs, although there are also unique proteins for apoptotic bodies, microvesicles and pEXOs, suggesting that the different EVs are involved in specific mechanisms. SNAs have been associated with the apoptosis process, as there is a hypothesis of STBs turnover that suggest effete nuclei are collected into knots, undergo apoptosis and shed into maternal bloodstream (Mayhew et al., 1999; Huppertz et al., 2002; Huppertz et al., 2003). However, other findings report that, despite nuclear condensation and signs associated to degeneration, it is unlikely that SNAs are related with apoptosis in non-pathological pregnancies (Coleman et al., 2013). SNAs have been observed after 6 weeks of pregnancy, suggesting a potential role in immune tolerance (Pantham and Chamley, 2018). SNAs have been divided in three categories: syncytial sprouts, knots, and bridges. Syncytial sprouts are pedunculated collections of euchromatic nuclei arising at the start of new villi and found during the first trimester. Knots appear at term, protruding slightly from the villous surface. Latelly, bridges are highly nucleated regions connecting two villi (Coleman et al., 2013). The function of SNAs remains to be revealed, but they appear to be engulfed by lung macrophages, and it is likely that they may lead to immunological silencing of these cells as shown for apoptotic bodies (Kupper and Huppertz, 2022).

## 3 Placental-derived extracellular vesicles in obstetric complications

In this section, a summary of the current knowledge of the role of PEVs in some of the most relevant obstetric diseases including preeclampsia (PE), gestational diabetes mellitus (GDM), fetal growth restriction (FGR), chorioamnionitis and preterm birth (PTB) is shown.

### 3.1 Preeclampsia

#### 3.1.1 Introduction

PE affects about 3% of all pregnancies and belongs to a group of diseases generically known as hypertensive disorders of pregnancy, together with chronic hypertension, gestational hypertension and chronic hypertension with superimposed PE (JA et al., 2011). High blood pressure is the main symptom of PE, but other symptons are frequent in this disease, such as proteinuria, edema, multiorgan failure, FGR and HELLP (Hemolysis, elevated liver enzymes, and low platelet count) syndrome and eclampsia. It has a fact that PE predisposes both the mother and the fetus to suffer cardiovascular diseases and other complications after birth (Jurewicz, 2018; Wallace et al., 2018)**.** Clinically, the diagnosis of PE is established from a systolic and diastolic blood pressure ≥140 mm Hg and ≥90 mm Hg, respectively, measured at least two times 4 h apart; higher values (≥160 mm Hg and ≥110 mm Hg, respectively) are frequent (Karrar and Hong, 2022). PE has been classified into mild or severe PE, although it is widely accepted that PE symptons can get worse very rapidly, posing a severe health problem for both the fetus and the mother (Tranquilli, 2013). However, it is common to distinguish between early onset PE (EO-PE) and late onset PE (LO-PE) depending on whether the appearance of the first symptoms takes place before or after the 34th week of gestation (Redman, 2017). The etiopathogenesis of EO-PE is unknown, although the most widely accepted hypothesis considers it consequence of a failure in the placentation process associated with defective spiral artery remodeling and trophoblast invasion. This leads to a persistent hypoxia/ischemia state in the placental tissue and to an imbalance in the angiogenic process which eventually leads to abnormal trophoblast behavior, oxidative stress, vascular inflammation and endocrine alterations. Eventually, systemic endothelial injury may lead to multiorgan failure and systemic complications (Ortega et al., 2022b). LO-PE is more likely bound to maternal extrinsic factors and, although placental damage also exists, the pathophysiological signatures, the impact of PE for both the fetus and mother, clinical features and serum markers are significantly different (Raymond and Peterson, 2011).

The only definite cure for PE is delivery, although low-dose aspirin (LDA) can be used as a prophylactic measure for patients at high risk (Bujold et al., 2010; Rolnik et al., 2017; Ives et al., 2020). Main risk factors associated to PE includes genetic background (family history) or previous events of PE; preexisting medical conditions, like hypertension, antiphospholipid syndrome, or insulin-dependent diabetes; obesity; age (≥40 years old), assisted reproductive techniques; nulliparity and multiparity (Lisonkova and Joseph, 2013; Paré et al., 2014). In this context and, in the absence of effective therapies, early prediction of PE is critical for preventive therapy and to guarantee adequate patient surveillance (Wagner, 2004; Sibai, 2005; Sunjaya et al., 2019). Likewise, a detailed understanding of the pathophysiology of PE will help to find novel biomarkers with translational applications.

#### 3.1.2 Role of placental extracellular vesicles in preeclampsia

PEVs are potential mediators of PE pathogenesis and offer promising therapeutic applications *from bench to bedside*. According to different research works, the number of EVs and PEVs are higher in preeclamptic women compared to unaffected women, suggesting a putative pathophysiological role of these components (Knight et al., 1998; Salomon et al., 2017). Likewise, PEVs are larger in PE women (Tong et al., 2017). Additionally, different studies have shown that there are differences between EO-PE and LO-PE, as the number of placental microvesicles and pEXOs is higher in EO-PE compared to LO-PE. However, some studies have described that there are not changes in the levels of PLAP + EV between normotensive and LO-PE women, confirming the etiopathogenic differences between EO-PE and LO-PE (Goswamia et al., 2006; Orozco et al., 2009; Chen et al., 2012; Pillay et al., 2016). Regarding their content, lipidomic analysis have revealed that STBMs from PE women display enhanced levels of total phosphatidylserine whereas phosphatidylinositol, phosphatidic acid, and ganglioside mannoside 3 were significantly downregulated in comparison with normal pregnancies (Baig et al., 2013).

The changes in the EV profile can critically affect to different pathophysiological mechanisms of PE. For instance, it has been demonstrated that administration of PEVs in a mice model of PE induced proteinuria and hypertension in non-pregnant mice, disrupting endothelial activity and promoting vasoconstriction (Han et al., 2020). Interestingly, the clearance of these pathological PEVs prevented the induction of the preeclampsia-like phenotype, suggesting a causal role of these components in PE. Gill et al., (2019) reported a remarkable increase of neprilysin (NEP) in PEVs obtained from PE women, playing pathological roles in hypertension, heart failure, and amyloid deposition, involved in the development of PE. Focusing on the vascular alterations and damage observed in the course of this obstetric disease Tannetta et al. (Tannetta et al., 2013) defined that there are differences in physical and antigenic features of PEV between normal and preeclamptic preparations. For instance, they showed that pEXOs and placental microvesicles presented lower PLAP levels in their surface and larger quantities of biologically active endoglin (Eng) and Fms Related Receptor Tyrosine Kinase 1 (Flt-1). Both Flt-1 and Eng as well as their soluble forms (sEng/sfl1-1) are major contributors to the pathophysiology of PE by reducing the bioavailability of the proangiogenic markers vascular endothelial growth factor (VEGF) and placental derived growth factor (PlGF) (Gilbert et al., 2008). SNAs appears to be a major source of sFlt-1 in PE, being associated with increased syncytial knots formation and shedding of these EV into maternal circulation and lungs and contributing to the systemic vascular injury observed (Rajakumar et al., 2012; Buurma et al., 2013). Besides, it has been described that macrovesicles from preeclamptic but not from normal placenta induced endothelial activation, suggesting a potential pathophysiological role EV in the endothelial injury systemically (Shen et al., 2014). Microparticles released by STBs seems to contain lower amounts of Tissue Factor Pathway Inhibitor (TFPI) enhancing the bioavailability of Tissue Factor (TF) and its procoagulant activity observed in women with gestational vascular complications (Aharon et al., 2009). Simultaneously, STBMs from PE women show higher levels of TF, consistently with the altered hemostasis reported in these patients (Gardiner et al., 2011). Also, PEVs from patients with PE contains higher levels of mucin-1, a glycoprotein which impairs EVT invasion *via* β1-integrin signaling (Shyu et al., 2011; Tannetta et al., 2017). Proteomic studies have evidenced decreased integrins in STBMs from PE women, related to reduced trophoblast invasion and defective placental vascularization (Baig et al., 2014). pEXOs can also present syncytin-1 and -2 in their membrane, two proteins involved in STB formation and critically downregulated in women with PE (Vargas et al., 2011). However, syncytin 2 is reduced in pEXOs from preeclamptic placenta when compared with normal women and there is a decreased internalization of exosomes released by trophoblast cells deficient in syncytin-1 and -2 (Vargas et al., 2014).

As above mentioned, an exacerbated inflammatory response and ischemia/hypoxia are important pathophysiological mechanisms in PE. Exosomes from EVT cultured *in vitro* under low oxygen tension increase TNFα expression in human umbilical vein endothelial cells (HUVECs), inhibiting their migration (Truong et al., 2017). Likewise, low oxygen tension was also associated with a differential miRNA signature in exosomal derived from EVT, all of them associated with cell migration and cytokine production (Truong et al., 2017). Shen et al., (2018) found that placenta-associated serum exosomal miR-155, which was upregulated in patients with PE, decreased nitric oxide (NO) production and endothelial nitric oxide synthase (eNOS) expression in primary HUVECs. Similarly, it has been reported (Ospina-Prieto et al., 2016) that increased levels of exosomal miR-141 derived from placental trophoblast in patients with PE could induce T cell proliferation. Kovacs et al. (Kovács et al., 2018) found that EV from PE women induce an aberrant response in macrophages, affecting chemotaxis, cell adhesion and migration *in vitro.* These authors described increased CD47 levels (“do not eat me” signal) and decreased external phosphatidylserine levels (“eat me” signal) along with a decreased uptake of these EVs. A possible explanation is partially described in other article (Wang et al., 2019), where the authors showed that miR‐548c‐5p expression was lower in serum exosomes and placental mononuclear cells in PE patients compared to non-pathological pregnancies. This down‐regulation is associated with an increased secretion of inflammatory cytokines (IL‐12 and TNF‐α) and nuclear translocation of NF‐κB, leading to an aberrant macrophage response. In addition to macrophages, neutrophils are also tightly modulated by PEVs. Indeed, *in vitro* studies have shown that PEVs can dramatically increase the production of superoxides by donor neutrophils and stimulate the production of neutrophil extracellular traps (NETs), reducing maternal blood flow in preeclamptic placentas (Tong and Chamley, 2015). Reduced levels of gelsolin reported in women with PE can be associated with an aberrant shedding of EVs while treating placental explants with preeclamptic sera enhanced expression of the proinflammatory marker High Mobility Group Box 1 (HMGB1) detected in SNAs, according to previous works (Zhang et al., 2020). Moreover, microvesicles derived from preeclamptic placenta exacerbated *in vitro* the response of PBMCs to LPS, supporting a proinflammatory action of these EVs in PE (Holder et al., 2012). Additional works have reported a reduced presence of the Placental Protein 13 (PP13) in PEV of PE women, suggesting that this dysregulation can be involved in the immunopathological mechanisms observed (Sammar et al., 2018)**.**

**FIGURE 2:**
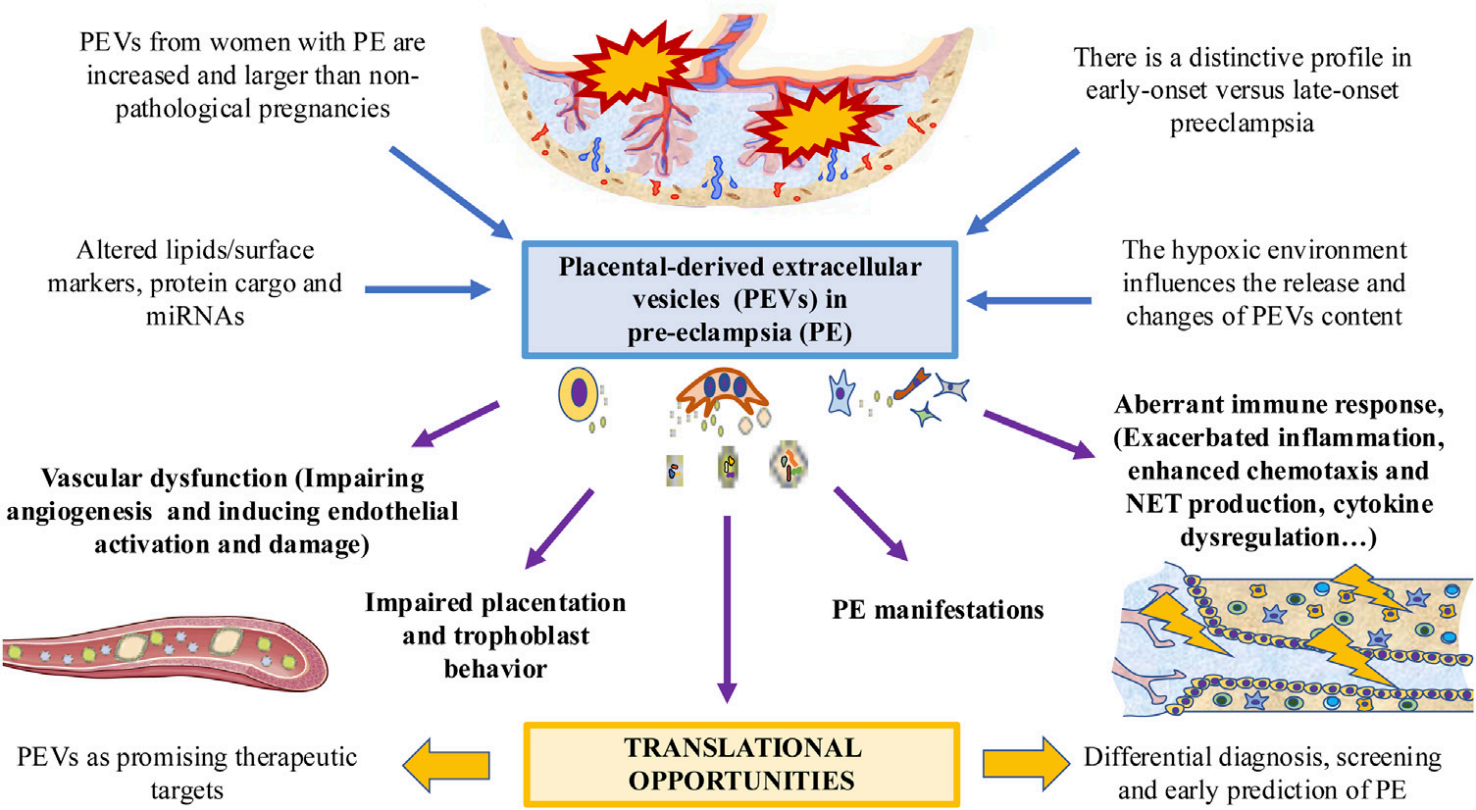
The role of PEVs in PE. PEVs are increased in women affected with this condition, with multiple changes in their surface and cargo. These changes are different in early *versus* late-onset PE. A possible modulator of PEVs is the low oxygen tension and hypoxic environment. PEVs released by the placenta participates in the PE-associated vascular dysfunction, impairing placentation and trophoblast behavior, dysregulating the immune system and leading to different PE manifestations. This can arise potential translational opportunities for exploring.

The diagnostic value of PEVs has also been tested. For example, Salomon et al. (Salomon et al., 2017) found that in asymptomatic women who lately developed PE, the concentration of total exosomes and pEXOs was higher than in controls. Similarly, hsa-miR-486-1-5p, and hsa-miR-486-2-5p miRNAs contained in the exosomes are elevated in PE compared with normal pregnancies, suggesting their potential application for the early PE biomarkers. Other authors (Biró et al., 2017) observed circulating exosomal total-miRNA and hsa-miR-210 levels were significantly elevated in PE women in comparison with normotensive women and patients diagnosed with other hypertensive disorders of pregnancy (chronic and gestational hypertension), suggesting a promising role for differential diagnosis. Some studies have described that apoptotic microparticles derived from trophoblast in PE women revealed a significant increase of circular DNA in comparison to normal pregnancies, which can be used for both early genetic diagnosis and monitoring of pathological pregnancies (Orozco et al., 2008). As summarized in Figure 2, the amount and content of PEVs is involved in the pathogenesis of PE, modulating several processes, such as the immune response, the angiogenic balance and other mechanisms related with this disease.

### 3.2 Gestational diabetes mellitus

#### 3.2.1 Introduction

GDM represents the most common medical complication of pregnancy The incidence and prevalence of this condition is increasing due to improved clinical diagnosis and screening programs (Alfadhli, 2015). The main risk factors for GDM are obesity, sedentary lifestyle, multiparity, family history of type 2 diabetes mellitus (T2DM), past history of GDM or delivery of a macrosomic baby, advanced maternal age, ethnicity and polycystic ovarian syndrome (Lin et al., 2016; Yaping et al., 2022). The main symptom associated with GDM is the appearance of chronic hyperglycemia during pregnancy without a previous diagnosis of diabetes (Diabetes Care, 2018). From a pathophysiological perspective, GDM is the result of pancreatic β-cell dysfunction on a background of chronic insulin resistance during pregnancy. In a healthy pregnancy, peripheral insulin sensitivity increases in early pregnancy, promoting the glucose uptake by adipose tissues for late pregnancy. Throughout the pregnancy, the pancreas undergoes a compensatory response with hyperplasia and hypertrophy to meet the energetic demands of this period. This leads to a decreased peripheral insulin sensitivity and increased blood glucose levels to be exchanged through the placenta and used by the fetus. After delivery, β-cell function, glucose levels and insulin sensitivity are restored (Catalano et al., 1991; Di Cianni et al., 2003). However, in GDM there is an ineffective compensatory response of the pancreas, that combined with reduced insulin sensitivity results in a marked hyperglycemia. It has been hypothesized that GDM women may have slightly impaired the peripheral insulin sensitivity before pregnancy and after delivery, insulin sensitivity, β-cell function and glucose levels can be either restored or might remain impaired on a pathway toward GDM in future pregnancy or T2DM (Plows et al., 2018). During the course of this complex disorder, there are several organs directly involved such as the brain, gut, liver, muscles and adipose tissue. In addition to the pancreas, the placenta is the most important organ involved in the pathogenesis of GDM. More specifically, the release of several hormones produced by the placenta (mainly lactogen, but also growth hormone, prolactin, corticotropin-releasing hormone and progesterone) promote insulin resistance and hyperglycemia (Rodriguez and Mahdy, 2022).

GDM is associated to multiple complications during pregnancy, increasing the incidence of hypertensive disorders of pregnancy like preeclampsia, polyhydramnios, excessive fetal growth, maternal and neonatal morbidities, including cesarean, neonatal hypoglycemia, hypocalcemia and respiratory stress syndrome, amongst others (Metzger et al., 2008). Long-term complications include increased risk of diabetes and cardiovascular disease in the mothers and obesity and diabetes for the children (Ashwal and HodGestational, 2015). According to the International Association Diabetes Pregnancy Study Groups (IADPSG) (Metzger et al., 2010), detection and diagnosis of GDM is usually performed by an oral glucose tolerance test (OGTT), considering the following criteria: 1) Measure of fasting plasma glucose (FPG), glycosylated hemoglobin (HbA1c), or random plasma glucose on all or only high-risk women; 2) If results not diagnostic of overt diabetes, GDM is clinically diagnosed when (FPG) ≥92 mg/dl and <126 mg/d; 3) if FPG is < 92 mg/dl, a 2-h 75-g OGTT should be performed at 24–28 weeks’ gestation after overnight fast, being diagnosed as GDM if FPG ≥92 mg/dl or 1 h plasma glucose ≥180 or 2-h plasma glucose ≥153. Changes in diet and physical activity are the primary treatments for GDM, but pharmacotherapy, usually insulin, is required when normoglycemia is not achieved. Oral hypoglycemic agents, principally metformin and glibenclamide (glyburide), can also be used (McIntyre et al., 2019).

#### 3.2.2 Role of placental extracellular vesicles in gestational diabetes

PEVs have been studied as well in the context of GDM pathogenesis. As mentioned before, glucose can stimulate the production and release of PEVs independently from oxygen tension (Rice et al., 2015). It has been reported GDM patients with 2.2-fold, 1.5-fold and 1.8-fold increased levels of PEVs with respect to normoglycemic pregnant women during the first, second and third trimester, respectively (Salomon et al., 2016)**.** The possible role of differences in proteins in PEVs isolated from GDM has been analyzed. Proteomic analysis of PEVs isolated at the time of GDM diagnosis showed differential expression of Calcium/calmodulin-dependent Protein Kinase II beta (CAMK2β) and Pappalysin-1 (PAPP-A), which are capable of influencing insulin signaling and glucose metabolic pathways (Jayabalan et al., 2019a). It has been reported that Dipeptidyl peptidase-4 (DPPIV) in GDM patients serum compared with normoglycemic pregnant women show greater DPPIV activity and a 8-fold increase of DDPIV-bound PEV (Kandzija et al., 2019). Importantly, DDPIV is characterized by breaking down glucagon-like peptide-1 (GLP-1), a critical regulatory molecule on glucose-dependent insulin secretion.

PEV-associated miRNAs have been studied as well in the context of GDM. Nair et al. (Nair et al., 2021a) described significative changes between normoglycemic and GDM women in 101 EV-associated miRNAs, suggesting an adaptative response to this pathological condition. Likewise, it has been described a significant increase in early pregnancy (6–15 weeks) in the expression of several EV-miRNAs and PEV-miRNAs such as miR‒122-5p, miR‒132-3p; miR‒1323; miR‒136-5p; miR‒182-3p; miR‒210-3p; miR‒29a-3p; miR‒29b-3p; miR‒342-3p, and miR-520h (Gillet et al., 2019). Similarly, PEVs seems to carry a set of miRNAs which modulates skeletal muscle insulin sensitivity. Consequently, PEVs isolated from women with GDM reduced insulin-mediated migration and glucose uptake in primary skeletal muscle cells from healthy individuals. Conversely, PEVs from normoglycemic women increased insulin-mediated migration and glucose uptake in primary muscle cells obtained from GDM patients (Nair et al., 2018). *In vivo* models also showed that PEV isolated from GDM women could induce impaired glucose tolerance and changes in the miRNA profile and insulin signaling in skeletal muscle tissues, specially through the phosphorylation of IRS-1 and Akt (James-Allan et al., 2020). Besides, when compared to normoglycemic PEVs, glucose stimulated insulin secretion from pancreatic islets were reduced.

**FIGURE 3:**
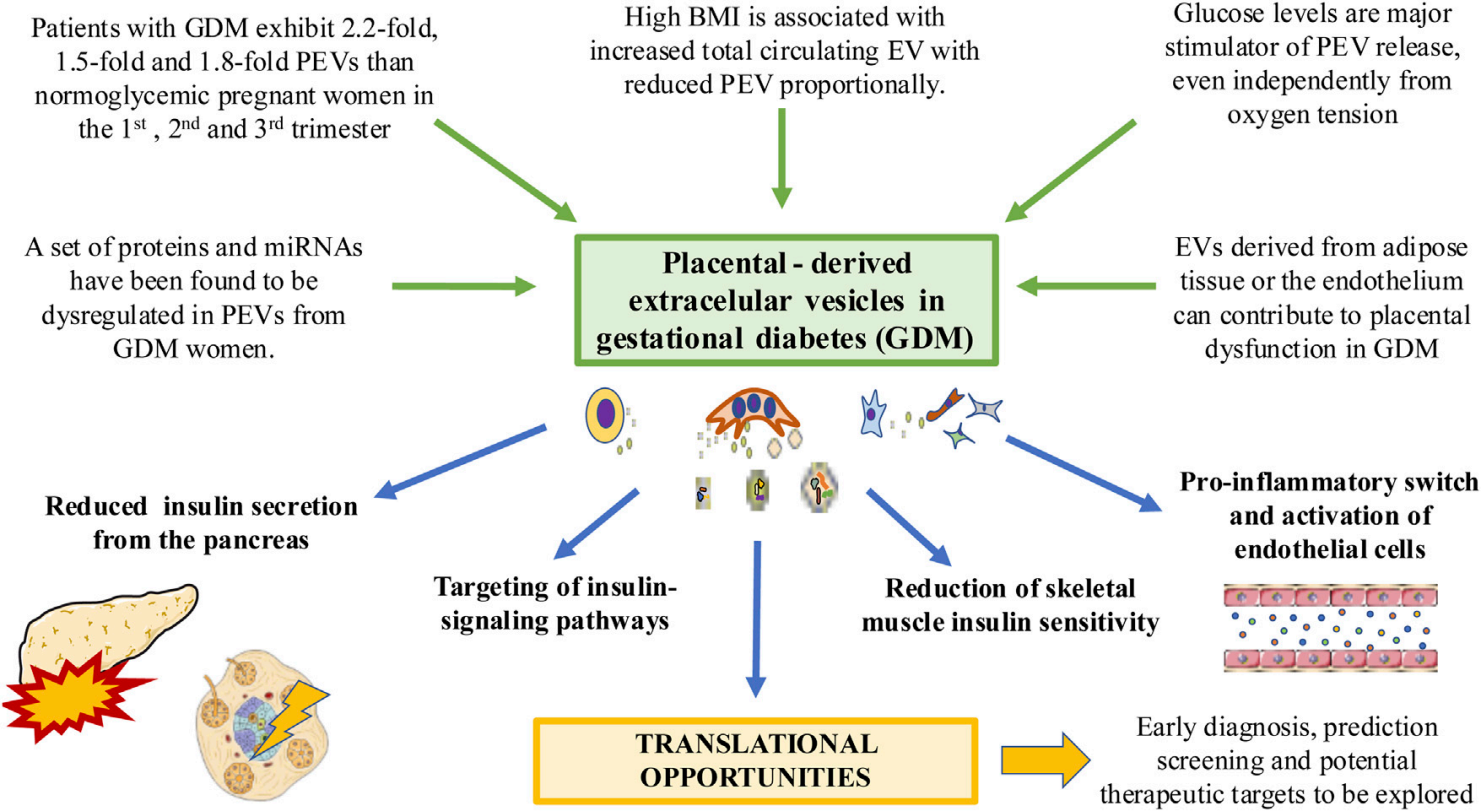
The relevance of PEVs in the context of GDM. Different factors associated with this condition such as hyperglycemia and high body mass index can induce significant changes in PEV profile. EVs from other tissues affected by GDM such as adipose tissue can induce changes in the placenta. PEVs can in turn reduce insulin secretion from the pancreas, altering insulin signaling pathways and insulin sensitivity in different tissues such as the muscle or endothelium, driving to its activation and pro-inflammatory switch. Translational knowledge will help to improve the clinical management of GDM patients.

There is a strong association of increased body mass index (BMI) values in early pregnancy (BMI>25) with GDM development (Zhang et al., 2022). Previous studies have described a positive correlation between BMI and total number of blood EV during pregnancy. However, the higher BMI is, the less is the number of PEVs to the total amount of EVs (Elfeky et al., 2017). Likewise, EVs from women with a BMI >25 display a proinflammatory profile, with an increased release of IL-6, IL-8 and TNF-α from endothelial cells (Salomon et al., 2016; Elfeky et al., 2017)**.** Previous studies have found that PEVs can stimulate the release of different proinflammatory cytokines by endothelial cells (Rice et al., 2015). In turn, EVs derived from the endothelium can promote significant pathophysiological changes related to GDM in the placenta (Sáez et al., 2018a; Sáez et al., 2018b) demonstrating the interaction that exist between PEVs and the endothelium. On the other hand, EVs produced by adipose tissue promote placental changes during GDM. In this sense, these EVs appears to be directly correlated with birthweight Z score, modulating different products and events like Sirtuin, oxidative phosphorylation, and mammalian target of rapamycin (mTOR) signaling pathway (Jayabalan et al., 2019b). Figure 3 summarizes the influence of PEVs in GDM. However, the effect of PEVs and EVs released by other tissues in GDM pathophysiology is far from being fully elucidated. The use of these PEVs as prognostic, predictive or diagnostic biomarkers as well as potential therapeutic targets is a promising field of study, with potential effects in the clinical management of these patients (Nair et al., 2021b).

### 3.3 Other pregnancy complications

In this section, we will review relevant findings in less prevalent obstetric complications in which PEVs have been implicated.

#### 3.3.1 Fetal growth restriction

Complications in maternal-fetal communication can lead to one of the most worrying pathologies in pregnancy, which is FGR. Traditionally, FGR describes a condition in which fetus is smaller (below the 10th percentile) than expected based on gestational age (SGA). However, as not every fetus has the same growth potential, so the definition has been expanded, and now it refers to those SGA fetuses due to a pathologic cause and consequently are at risk of adverse events (Easter et al., 2017). Current consensus requests weight <third percentile or three out of the following criteria: birth weight <10th percentile, head circumference <10th percentile, length <10th percentile, prenatal diagnosis of FGR, and the presence of any pregnancy complication (Beune et al., 2018). To diagnose FGR accurately, umbilical artery Doppler assessment must be abnormal and percentile limits are 10, 5 or 3 compared to a population of reference. Then subjacent causes must be detected. In developed countries, the frequency of FGR increases with the age of the mother and often is the consequence of preexisting preeclampsia but in developing countries malnutrition and infectious diseases are relevant factors (Easter et al., 2017). Other factors involved are maternal hypertension, diabetes mellitus, autoimmune diseases, smoking, or drug and alcohol abuse. Among the factors linked to the fetus chromosomal abnormalities (trisomy 13, 18, and 21), mutations at insulin-like growth factor or multiple gestation are the most relevant (Condrat et al., 2021). Inadequate placentation is also important. In all these cases, pathological extracellular vesicle trafficking at the feto-maternal crosstalk has been suggested by several authors.

In those cases of FGR underlying preeclampsia, the surface area available for feto-maternal traffic is decreased due to deficient remodeling of uterine spiral arteries and villous damage, reducing the exchange of oxygen and nutrients. Some prognostic biomarkers used in preeclampsia have been employed in FGR as well. Low levels of angiogenic molecules like PIGF or altered serum levels of PAPP-A in the first trimester are considered in clinical practice for prenatal screening. Low circulating levels of anabolic hormones IGF-1 and its binding proteins explain nutrient privation and reduced growth potential. Finally, circulating EGF Like Domain Multiple 7 (EGFL7) has been used to classify isolated FGR and preeclampsia at different gestational stages (Ortega et al., 2022b).

Predictive biomarkers for clinic practice are not available at the moment. Future studies will focus on the characterization of placenta and fetal-derived EVs and their cargoes to find predictive biomarkers (Buca et al., 2020). Prospective cohort studies studying placenta-derived exosomes in maternal and fetal circulation for fetuses with FGR or SGA have been published. In a study, the authors reported reduced [PLAP^+ve^:total exosomes] ratios in FGR compared to controls and SGA cases (Miranda et al., 2018). Likewise, EVs derived from umbilical cord as well as the expression levels of miR-150 either in tissue or in EV were decreased in a pig model of FGR. Upregulation of this miRNA promoted proliferation, cell migration and tube formation by HUVEC cells, promoting a pro-angiogenic effect (Luo et al., 2018). These results support the existence of a pathogenic feto-maternal communication and uteroplacental vascular insufficiency. Hromadnikova et al., (2019) in a retrospective case control study, selected C19MC miRNAs exosomes from early gestation stages, observing a down-regulation of miR-520a-5p in first trimester of women suffering preeclampsia and gestational hypertension who later developed FGR. These authors described this miRNA as a novel biomarker for the onset of FGR due to its abundance in placenta and reduced expression levels in other tissues. Similarly, Rodosthenous et al., (2017) noticed that the level of various EV-associated miRNAs in the second trimester of pregnancy were correlated with fetal growth, describing sex-specific associations. However, future studies are needed to unravel the role of EVs and PEVs in FGR.

#### 3.3.2 Infections

Chorioamnionitis (infection of the membranes and chorion of the placenta) represents a common complication of pregnancy associated with significant perinatal, maternal and long-term adverse consequences (Tita and Andrews, 2010). Chorioamnionitis can be acute, subacute or chronic, showing different clinical characteristics and complications (Fowler and SimonChorioamnionitis, 2022). Chorioamnionitis is classified in two main categories: Histologic (based on microscopic evidence of inflammation of the membranes) and clinical (based on medical manifestations of local and systemic inflammation such as fever, abdominal pain or leukocytosis (>15,000 cells/mm^3^) (Menon et al., 2010)**.** There are plenty etiological agents that may cause chorioamnionitis. The most frequent cause of chorioamnionitis are ascending bacteria from the vagina and cervix, appearing as a polymicrobial infection secondary complication of prolonged rupture of the membranes (Czikk et al., 2011). Other less common agents include viruses, fungi and even parasites like *Plasmodium falciparum* (Czikk et al., 2011; Singoei et al., 2021). Previous works have noticed an important role of PEVs in chorioamnionitis. As above mentioned, PEVs seem to have an impact in resistance pathways of viral infection. For instance, PEVs are able to inhibit viral replication, or they may content some antiviral agents like interferon (IFNλ1), although they can also favor viral spread, acting as a secure vehicle for them and promoting immune evasion (Condrat et al., 2021). Otherwise, PEVs also play a pivotal role in bacterial infections. Kaletka et al. (Zeldovich et al., 2013) studied trophoblastic EVs afer being infected by *Listeria monocytogenes*. Interestingly they found that these PEVs were immunostimulatory, activating macrophages to a proinflammatory state, but also making them more vulnerable to being infected by this bacteria. Thus, both effects are likely observed in PEVs regarding infections: Inducing an immune response but also, promoting the spread of the pathogens. Similarly, Bergamelli et al., (2021) demonstrated that infection with human cytomegalovirus (CMV) affected *in vitro* the expression level of several surface markers in PEVs, defining an altered profile of these components due to this infection. Although these changes can be useful for defending against the infection, a very recent article has demonstrated that PEVs from infected CMV potentiate infection in in naive recipient cells of fetal origin, including human neural stem cells (Bergamelli et al., 2022). Besides, the authors proposed PEVs as central players of viral dissemination to the fetal brain due to congenital CMV infection and this statement is supported by Gall et al., (2022) who recognized the critical role of PEVs in different neuroinflammatory processes and the development of perinatal brain injury in the setting of chorioamnionitis by propagating and sustaining the inflammatory cascade. On the other hand, Moro et al., (2016) studied placental MVs and their miRNA content in pregnant women with Human Immunodeficiency virus (HIV) and *Plasmodium falciparum* infection. Interestingly, they show that HIV-infected mothers exhibited higher concentrations of total and trophoblast microparticles, which induced a higher expression of MHCII and lower production of MCP1. On the other hand, placental malaria was characterized by an upregulated miR-517c, which might have a pathogenic role on the adverse outcomes during pregnancy and malaria infection. Overall, the role of PEVs in chorioamnionitis and infections show promising but still reduced results, and due to the relevance of placenta in these conditions, further efforts would be of great aid in this field.

#### 3.3.3 Preterm birth

PTB (characterized by labor prior to 37 completed weeks of pregnancy) affects approximately 15 million infants yearly, representing an important cause of neonatal morbidity and mortality (Blencowe et al., 2013). The etiopathogenesis of this condition remains to be fully described, but different risk factors such as high blood pressure, diabetes, obesity, underweight, psychological stress, history of previous preterm labor, exacerbated inflammation during pregnancy and tobacco smoking are implicated in the development of premature birth (Stewart and Graham, 2010; Condrat et al., 2021). PTB has been associated with higher number of EVs (Menon and Shahin, 2021) and *in vivo* models have shown that these EV could induce preterm labor due to the presence of increased inflammatory mediators (Sheller-Miller et al., 2019). Proteomic studies have identified 72 proteins that might play a crucial role in preterm labor, being associated with critical inflammatory and metabolic signals (Menon et al., 2019a). Similarly, Cantonwine et al., (2016) described that from a total of 62 proteins contained in circulating EVs qualified for diagnosis alpha-2-macroglobulin (α2M), human endogenous medium-reiteration-frequency-family-34 ORF (HEMO), and mannose binding lectin 2 (MBL2), displayed a specificity of 83% with median area under the curve (AUC) of 0.89, which could be use as predictive biomarkers of spontaneous PTB if validated in future studies. Simultaneously, Menon et al., (2019b) described up to 173 miRNAs importantly altered in serum exosomes of women with PTB. However, they could not describe the precise origin of these exosomes. Likewise, a possible role of bacterial exosomes derived from Ureaplasma and Veillonellaceae are more abundant in the urine of women with PTB (Yoo et al., 2016) suggesting the complex background of EVs in PTB.

**FIGURE 4:**
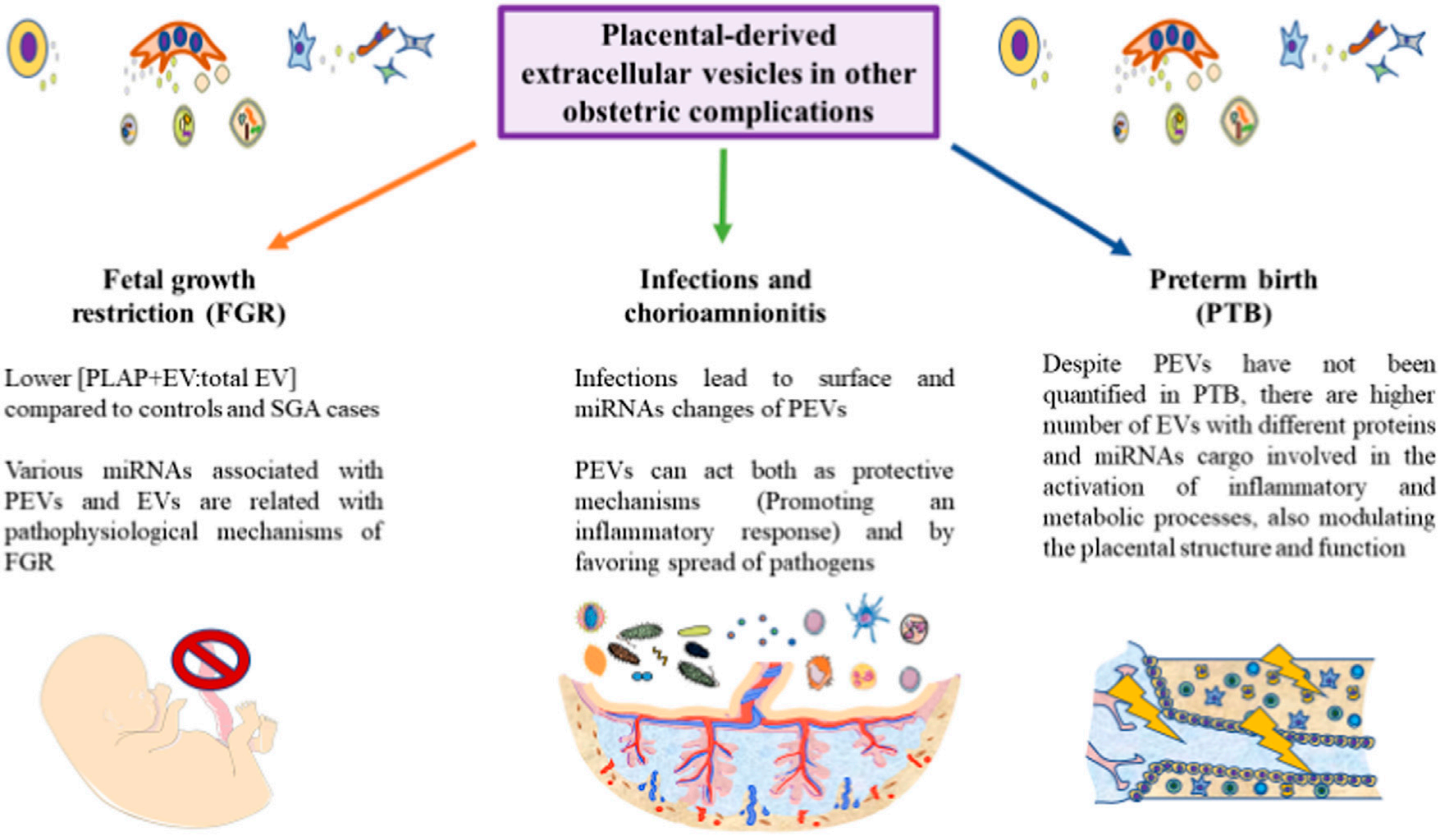
A graphical abstract of some of the current notions of PEVs in different obstetric pathologies. As represented, the role of PEVs remains to be further explored in the field of fetal growth restriction, infections and preterm birth. However, some initial results are starting to arise in these conditions, aiding to improve our knowledge of these obstetric complications. Due to the relevance of PEVs in many physiological and pathological processes, we encourage for future studies in these areas.

Although less studied PEVs have also been identified as critical mechanisms involved in the onset and progression of labor, having been suggested as potential biomarkers for preterm delivery (Salomon et al., 2018). The amount of PEVs in PTB has not been explored; however, there is a clinical case in which a dramatic increase of PLAP (10.5-fold), which can be present in PEVs, was associated with PTB (Ferianec and Linhartova, 2011). Fallen et al. (Fallen et al., 2018) identified a set of circulating and EV-associated miRNAs with potential pathophysiological effects in the placenta associated to PTB. Due to the multiple roles of the placenta during pregnancy and labor, we encourage for specific studies that focus on PEV in PTB, as this might be used as promising biomarkers in this research area. In Figure 4, the main implications of PEVs described until date is summarized.

## 4 Conclusion

The placenta is an active organ fulfilling multiple functions in pregnancy, both at physiological and pathological conditions. EVs has received growing attention in the last decade, acting as vehicles of a wide variety of molecules and signals involved in cell-to-cell communication. PEVs (pEXOs, placental microvesicles and apoptotic bodies) are a subgroup of EV which has been increasingly explored in the placentation process and throughout normal pregnancy, interacting with the maternal and fetal tissues. Likewise, the alteration of PEVs in different obstetric complications have become a potential field of study in recent years. Hence, changes in the concentration and cargo of PEVs have been implicated in the development of different pathologies like PE, GDM, FGR and so on. In Table 1, some of the most relevant findings regarding the molecular content of PEVs and their physiological/pathological role are summarized. However, further studies are needed to unravel the implications of these PEVs, developing more accurate methods to isolate and handle with these vesicles, disentangling the specific content in their surface and inward, as well as the translational applications derived from the basic knowledge.

**TABLE 1:** A summary of the main molecular findings and their potential implications in PEVs.

Molecular cargo	Type of PEV	Physiological functions/Pathological role	References
(HLA)-G5, B7-H1 and B7-H3	Placental exosomes	Immune tolerance. Dysregulation of HLA components are related to different obstetric complications	Kshirsagar et al. (2012)
miR-29a-3p	Placental exosomes	Enhanced expression of PDL-1 and M2 polarization in normal pregnancies	Bai et al. (2022)
NKGD2 ligands (i.e. ULBP and MIC-A and B	Placental exosomes	Immunosupressive role on NK cells	Hedlund et al. (2009)
miR-517a-3p	Placental exosomes	Targeting of PRKG1 in NK cells	Kambe et al. (2014)
PDL-1, PDL-2, TRAIL and FasL	Placental exosomes	Immunomodulatory effects in different populations	Mincheva-Nilsson, (2021)
TGF-β1 and IL-10	Placental microvesicles	Modulation of caspase 3 activity in CD56^dim^ NK cells	Nardi et al. (2016)
IL-1β	Placental microvesicles	More abundant in placental microvesicles during the first trimester or under pathological conditions	Holder et al. (2012)
miRNA members of the chromosome 19 miRNA cluster	Placental exosomes	Attenuate viral replication in recipient cells by the induction of autophagy	Delorme-Axford et al. (2013); Bayer et al. (2015))
PLAP	Placental exosomes and MVs	Marker of PEVs; Transfer of maternal IgG to the fetus at the placenta surface and stimulate DNA synthesis and cell proliferation in fetal fibroblasts. There is a direct correlation between (PLAP + ve ratio) and birth weight percentile. Reduced PLAP + ve ratio is associated with different obstetric complications. A single case report found a dramatic increase of PLAP associated with preterm birth	Dragovic et al. (2015); Jin and Menon, (2018); Miranda et al. (2018)
			Goswamia et al. (2006); Orozco et al. (2009); Chen et al. (2012); Pillay et al. (2016)
			Gilbert et al. (2008)
			Miranda et al. (2018)
			Ferianec and Linhartova, (2011)
Eng and Flt-1	Placental exosomes and MVs	Reduced bioavailability of VEGF abnd PlGF in preeclampsia	Tannetta et al. (2013)
sFlt-1	Syncytial nuclear aggregates	increased syncytial knots and systemic vascular injury in preeclampsia	Rajakumar et al. (2012); Buurma et al. (2013)
TFPI	Microvesicles	Enhanced bioavailability of TF and procoagulant activity in women with preeclampsia	Aharon et al. (2009)
TF	Syncytiotrophoblast microvesicles	Altered hemostasis in preeclamptic women	Gardiner et al. (2011)
mucin-1	Not specified	Impairs EVT invasion and integrin signaling in preeclampsia	Shyu et al. (2011); Tannetta et al. (2017)
Integrins	Syncytiotrophoblast microvesicles	Decreased in preeclampsia	Baig et al. (2014)
syncytin- 1 and -2	Placental exosomes	Syncytin 2 is decreased in placental exosomes of women with preeclampsia, affecting to their internalization	Vargas et al. (2011); Vargas et al. (2014)
miR-155	Placental exosomes	Decrease NO production and eNOS in primary HUVECs	Shen et al. (2018)
miR-141	Placental exosomes	Augmented in preeclampsia; Induction of T cell proliferation	Ospina-Prieto et al. (2016)
miR‐548c‐5p	Placental exosomes	Downregulation of expression of this miRNA alters macrophage behavior and promote the expression of the proinflammatory cytokines IL-12, TNF‐α and nuclear translocation of NF‐κB	Wang et al. (2019)
HMGB1	Syncytial nuclear aggregates	Increased in preeclampsia Proinflammatory actions in preeclamptic women	Zhang et al. (2020)
PP13	Not specified	Reductions of this protein is associated with aberrant immunopathological mechanisms	Sammar et al. (2018)
hsa-miR-210, hsa-miR-486-1-5p and hsa-miR-486-2-5p, circular DNA	Placental exosomes	Elevated in preeclampsia, being evaluated as potential diagnostic markers	Gillet et al. (2019)
CAMK2β, PAPP-A	Not specified	Insulin signaling and glucose metabolic routes	Jayabalan et al. (2019a)
DDPIV		Greater levels and activity in placental extracellular vesicles in women with gestational diabetes, modulating glucose-dependent insulin secretion	Kandzija et al. (2019)
miR-520a-5p	Not specified	Downregulated in women suffering preeclampsia and gestational hypertension and who later developed FGR	Hromadnikova et al. (2019)
